# Long non-coding XIST *raises* methylation of TIMP-3 promoter to regulate collagen degradation in osteoarthritic chondrocytes after tibial plateau fracture

**DOI:** 10.1186/s13075-019-2033-5

**Published:** 2019-12-09

**Authors:** Hongwei Chen, Shengdi Yang, Ruyi Shao

**Affiliations:** 1 Department of Orthopedic Surgery, Yiwu Central Hospital, the Affiliated Yiwu Hospital of Wenzhou Medical University, Yiwu, 322000 People’s Republic of China; 2 Department of Hand-Foot Microsurgery, Lanshi Hospital, Lanzhou, 730050 People’s Republic of China; 3 Department of Orthopedics, Zhuji People’s Hospital, No. 9, Jianmin Road, Zhuji, 311800 Zhejiang Province People’s Republic of China

**Keywords:** Long noncoding RNA XIST, TIMP-3, Methylation, Osteoarthritis, Chondrocytes, Collagen degradation, Tibial plateau fracture

## Abstract

**Background:**

Hypermethylation of gene promoters has been regarded as an epigenetic regulator for gene inactivation in the development of several diseases. In the current study, we aimed to explore how long noncoding RNA X-inactive specific transcript (lncRNA XIST) function in collagen degradation in chondrocytes of osteoarthritis (OA) after tibial plateau fracture by regulating tissue inhibitor of metalloproteinase-3 (TIMP-3) promoter methylation.

**Methods:**

In silico analysis was used to screen differentially expressed lncRNAs in cartilage tissues of OA. Chondrocytes were then successfully isolated from normal and OA cartilage tissues and identified, with the expressions of lncRNA XIST and TIMP-3 examined. The methylation levels of TIMP-3 promoter were determined by MS-PCR. The binding of lncRNA XIST to DNA methyltransferase and the binding of TIMP-3 promoter to DNA methyltransferase were determined by a series of experiments, including RIP, RNA pull-down, and ChIP assays.

**Results:**

The differentially expressed lncRNA XIST was determined in OA. In addition, cartilage tissues of OA showed upregulation of lncRNA XIST and downregulation of TIMP-3. LncRNA XIST was primarily localized in the nucleus and was capable of binding to the promoter of TIMP-3. The silencing of lncRNA XIST decreased the methylation levels of TIMP-3 promoter and increased the expressions of TIMP-3, which consequently inhibited collagen degradation in OA chondrocytes. Furthermore, TIMP-3 over-expression reversed the effect of lncRNA XIST on collagen degradation in OA chondrocytes.

**Conclusion:**

Collectively, lncRNA XIST raises collagen degradation in OA chondrocytes after tibial plateau fracture by accelerating the methylation of TIMP-3 promoter by recruiting DNA methyltransferase.

## Background

Tibial plateau fractures fall into the class of traumatic injuries and usually have a bimodal distribution with low-energy injuries that occur in the elderly population and high-energy injuries among the younger population [1]. Osteoarthritis (OA), the most common form of arthritis [2], is well-acknowledged as a common sequela of severe articular fracture, especially involving the weigh-bearing joint [3]. OA is characterized by the progressive breakdown of articular cartilage along with remodeling of the underlying bone in synovial joints and generally affects the spine, hips, knees, and fingers [4]. Chondrocytes are the only cell types that participate in the formation of the articular cartilage, whose progressive degradation has been found to be the primary cause of OA [5]. The normally practiced therapeutic regimens for OA include pain management, physical therapy, steroids, and other anti-inflammatory drugs, among which joint replacement is considered as the final option [6]. However, the risk of complications and critical recovery periods has been taken into consideration to achieve success in various surgical modalities for patients with OA [7]. In order to avoid these risks and complications, there is a need to develop other novel therapeutic methods that will work on eliminating the causative mechanism of OA.

OA occurs as a result of consistent inflammatory processes in the cartilage, accompanied by cell apoptosis [8]. Numerous researches have indicated that long noncoding RNAs (lncRNAs) are involved in cellular physiology. For example, lncRNA X-inactive specific transcript (lncRNA XIST), a member of the first discovered cancer-related lncRNAs, has been implicated in the progression of multiple types of tumors [9]. An additional correlation has also been identified between lncRNAs and cartilage injury and the degradation of chondrocytes [10, 11]. Cartilage samples obtained from OA patients were found to exhibit high expressions of lncRNA XIST [12]. The in silico analysis at the initial stage of our study further revealed a high expression of lncRNA XIST in OA tissues relative to its normal counterparts. Moreover, there is a strong expression of metalloproteinases (MMPs) in articular chondrocytes, which can degrade collagen and destruct the cartilage [13]. Tissue inhibitor of metalloproteinase 3 (TIMP-3) is an inhibitor of MMPs [14]. A previous study demonstrated that TIMP-3 is poorly expressed in condyle cartilages during the early stages of OA [15]. Recent researches have further reported that there is a significant difference in DNA methylomes when comparing normal patients with OA patients, and the gene expression involved in OA pathogenesis can be regulated by DNA methylation [16–18]. The aforementioned evidences indicate that lncRNA XIST and TIMP-3 may participate in the development of OA with the involvement of DNA methylation. Therefore, we hypothesized there might be a relationship between the expression of lncRNA XIST and TIMP-3 and collagen degradation in OA patients.

## Materials and methods

### Ethics statement

The current study was approved by the Institutional Review Board of Yiwu Central Hospital, the Affiliated Yiwu Hospital of Wenzhou Medical University and conducted in strict accordance with Declaration of Helsinki (Clinical trial registration number: ChiCTR1900025348). Signed informed consents were obtained from all patients prior to the study.

### Microarray-based analysis

Firstly, OA microarray data (GSE51588) were retrieved from the Gene Expression Omnibus database (https://www.ncbi.nlm.nih.gov/geo/) [19]. The “limma” package of R language (http://master.bioconductor.org/packages/release/bioc/html/limma.html) was applied to conduct differential analyses of gene expression [20]. In addition, |log Fold Change| > 2 and *p* value < 0.05 were set as the screening criteria for differentially expressed genes (DEGs). Subsequently, the “pheatmap” package of R language was used to plot a heatmap depicting the expression of DEGs.

### Study subjects

Cartilage tissues were obtained from OA patients who underwent surgical procedures for tibial plateau fracture at the Yiwu Central Hospital, the Affiliated Yiwu Hospital of Wenzhou Medical University from July 2016 to December 2017. A total of 15 OA cartilage specimens were collected from the patients and then assigned as the OA group, including 6 males and 9 females, aged 36–52 years with a mean calculated age of 45.20 ± 4.54 years. The samples were collected within 2 weeks following the diagnosis of OA in these patients for subsequent experiments. In addition, 7 samples of normal cartilage tissues were obtained from non-OA patients following amputation due to trauma and were regarded as the normal group. The collected cartilage tissues were stored at − 80 °C, a portion of which were fixed with 10% formalin and preserved in paraffin.

### Chondrocyte culture

The cartilage tissues collected from normal controls and patients with OA were subjected to cell isolation and culture. Next, the cartilage tissue samples were ground into 1–5-mm^3^ sections using a scalpel. The cartilage sections were then detached with 0.2% collagenase II (5–8 mL; Sigma-Aldrich, St. Louis, MO, USA) for 12–16 h at 37 °C with 5% CO_2_ in air, followed by supplementation of Dulbecco’s modified Eagle’s medium (DMEM)/F12 (HyClone, Logan, UT, USA) to stop the process of detachment. Following centrifugation at 150×*g* for 6 min, the chondrocytes released at the bottom of the centrifuge tube were absorbed into a culture bottle. Subsequently, the chondrocytes were cultured in 5 mL DMEM/F12 containing 15% fetal bovine serum (FBS) (Gibco, Grand Island, NY, USA) and 1% penicillin/streptomycin solution (Gibco, Grand Island, NY, USA) and incubated in a plastic culture bottle at 37 °C for 24 h with 5% CO_2_ in air. The adherent cells were cultured in a bottle for 2 weeks, and the fresh culture medium was renewed every 3 days before the cells were subcultured. Then the subcultured chondrocytes were plated in six-well culture plates and serum starved for 24 h with DMEM/F12 medium containing 1% FBS to synchronize the cells in a non-activating and non-proliferating phase. The chondrocytes were subsequently cultured again in DMEM/F12 containing 15% FBS [21].

### Toluidine blue staining for morphological identification of chondrocytes

The chondrocytes were inoculated into a six-well plate, and when the cells reached 50–60% confluence, the culture medium was discarded. The chondrocytes were then fixed in 4% paraformaldehyde for 30 min, stained with 1% toluidine blue at room temperature for 10–30 min, washed with absolute ethyl alcohol until they were colorless, and observed under an inverted microscope (Olympus, Tokyo, Japan). The nuclei were stained as blue by 4′,6-diamidino-2-phenylindole (DAPI).

### Immunocytochemistry

Once the migrated cells fully covered the coverslip, the coverslip was taken out and fixed using the same method as toluidine blue staining. Following 10-min incubation at room temperature with 3% H_2_O_2_, the samples were rinsed with phosphate buffer saline (PBS), blocked with normal non-immune animal serum, and incubated at room temperature for 10 min. Following the removal of the serum, the sample was incubated with primary rabbit polyclonal antibody to type II collagen (dilution ratio of 1:500, ab34712, Abcam Inc., Cambridge, UK) at 4 °C overnight, followed by a PBS rinse. Next, the biotin-labeled secondary antibody was added for a 10-min incubation at room temperature. After a PBS rinse, diaminobezidin was added for coloration, followed by hematoxylin counterstaining and hydrochloric acid alcohol differentiation. Then, the samples were dehydrated using absolute ethanol. The neutral balsam-sealed samples were observed under an inverted microscope and photographed. The positive cells were identified as those with yellowish-brown cytoplasm.

### Cell treatment

The chondrocytes included normal chondrocytes and OA chondrocytes. The OA chondrocytes were transfected with pGPU6/Neo-XIST plasmid [the short hairpin (sh)-XIST group, silencing of lncRNA XIST], pGPU6/Neo plasmid [the sh-negative control (NC) group, NC of lncRNA XIST silencing], pCMV6-AC-GFP-XIST plasmid [the over-expression (oe)-XIST group, over-expression of lncRNA XIST] pCMV6-AC-GFP-TIMP-3 plasmid (the oe-TIMP-3 group, over-expression of TIMP-3), pCMV6-AC-GFP plasmid (the oe-NC group, NC of lncRNA XIST over-expression), pCMV6-AC-GFP-XIST + pCMV6-AC-GFP plasmid (the oe-XIST + oe-NC group, over-expression of lncRNA XIST and NC of TIMP-3 over-expression), and pCMV6-AC-GFP-XIST + pCMV6-AC-GFP-TIMP-3 plasmid (the oe-XIST + oe-TIMP-3 group, over-expression of both lncRNA XIST and TIMP-3), respectively. The pCMV6-AC-GFP and pGPU6/Neo plasmids were purchased from Wuhan Miaoling Biotechnology Co., Ltd., (Wuhan, Hubei, China) and GenePharma Co., Ltd. (Shanghai, China), respectively. The chondrocytes were plated into 12-well plates at a density of 3 × 10^5^ cells/well. Lipofectamine 2000 kits (11668019, Thermo Fisher Scientific, Waltham, UK) were applied to transduce the plasmids into the chondrocytes when cells reached 80% confluence. Next, 4 μg target plasmid and 10 μL Lipofectamine 2000 were diluted in 250 μL serum-free Opti-MEM medium (Gibco, Grand Island, NY, USA), respectively. The above products were mixed after 5 min and incubated at room temperature for 20 min, which were then added into the wells, followed by culture in a humidified incubator at 37 °C with 5% CO_2_ in air. After 6 h, the culture medium was replaced with the complete medium. After an additional 72 h of culture, the cells were collected for subsequent experimentation.

### Reverse transcription quantitative polymerase chain reaction (RT-qPCR)

The extraction of total RNA from tissues and cells was performed with the use of TRIzol (Invitrogen, Carlsbad, CA, USA), and the concentration and purity of total RNA were determined by a nanodrop2000 microultraviolet spectrophotometer (1011U, Nanodrop technologies, Wilmington, DE, USA). The complementary DNA (cDNA) was obtained based on the instructions of the PrimeScript RT reagent Kit (RR047A, Takara, Tokyo, Japan). Primers of lncRNA XIST and TIMP-3 were designed and synthesized by TaKaRa Biotechnology Co., Ltd. (Dalian, Liaoning, China) (Table 1). RT-qPCR was performed using ABI7500 qPCR instrument (7500, Applied Biosystems, Carlsbad, CA, USA). With glyceraldehyde phosphate dehydrogenase (GAPDH) as the internal control, the relative transcription level of the target gene was calculated using the 2^−△△CT^ method [22]. The experiment was conducted in triplicates.

**Table 1:** The primer sequence for RT-qPCR

Gene	Primer sequence (5′–3′)
LncRNA XIST	F: 5′-GACACAAGGCCAACGACCTA-3′
	R: 5′-TCGCTTGGGTCCTCTATCCA-3′
TIMP-3	F: 5′-ATCTCCCAGACCCTCTTCCC-3′
	R: 5′-AGTCTGGTTGGGACATGCAG-3′
GAPDH	F: 5′-CCATGTTCGTCATGGGTGTGAACCA-3′
	R: 5′-GCCAGTAGAGGCAGGGATGATGTTC-3′

Notes: *F* forward, *R* reverse, *LncRNA* long noncoding RNA, *XIST* X-inactive specific transcript, *TIMP-3* tissue inhibitor of metalloproteinase 3, *GAPDH* glyceraldehyde phosphate dehydrogenase, *RT-qPCR* reverse transcription quantitative polymerase chain reaction

### Western blot analysis

Total protein content was extracted from tissues and cells by radioimmunoprecipitation assay (RIPA) containing phenylmethylsulfonyl fluoride (PMSF) and incubated on ice for 30 min. The supernatant was collected after centrifugation at 8000×*g* at 4 °C for 10 min. The protein concentration was determined with using bicinchoninic acid (BCA) kits, and the proteins were separated with sodium dodecyl sulfate polyacrylamide gel electrophoresis. Afterwards, the proteins were transferred onto a polyvinylidene fluoride membrane, which was sealed with 5% skimmed milk for 1 h at room temperature. The primary rabbit antibodies against TIMP-3 (dilution ratio of 1:1000, ab39184), matrix metalloproteinase-3 (MMP-3) (dilution ratio of 1:1000, ab53015), MMP-13 (dilution ratio of 1:3000, ab39012), A disintegrin and metalloproteinase with thrombospondin motifs (ADAMTS)-4 (dilution ratio of 1:2000, ab185722), ADAMTS-5 (1:250, ab41037), type II collagen (dilution ratio of 1:5000, ab34712), and GAPDH (dilution ratio of 1:2500, ab9485) were added onto the membrane for incubation at 4 °C overnight. The aforementioned antibodies were all purchased from Abcam Inc. (Cambridge, UK). The membrane was then rinsed three times with Tris-buffered saline Tween-20 (TBST). Then, the horseradish peroxidase (HRP)-labeled secondary antibody of goat anti-rabbit antibody against immunoglobulin G (IgG) (dilution ratio of 1:2000, ab97051, Abcam Inc., Cambridge, UK) was added onto the membrane for further incubation for 1 h, followed by development using enhanced chemiluminescence detection kits (BB-3501, Amersham Biosciences, Bucks, UK). The Bio-Rad image analysis system (Bio-Rad, Hercules, CA, USA) was adopted in order to obtain the images, and the Quantity One v4.6.2 software was applied for protein band analysis. The relative protein expression was calculated as the ratio of gray values of the target protein band to that of GAPDH protein band. The experiment was conducted in triplicates to obtain the mean value.

### Fluorescence in situ hybridization (FISH)

The FISH technique was employed to identify the subcellular localization of lncRNA XIST in OA chondrocytes, which was conducted based on the instructions provided by the Ribo™ lncRNA FISH probe Mix (Red) (RiboBio, Guangzhou, Guangdong, China). A cover glass was placed onto the six-well plates, and the chondrocytes were seeded into plates until the cell confluence reached 80%. Subsequently, the cover glass was removed, fixed with 1 mL 4% paraformaldehyde at room temperature, and treated with protease K (2 μg/mL), glycine, and reagent for acetylation. The chondrocytes were then processed with 250 μL prehybridization solution and incubated at 42 °C for 1 h. The prehybridization solution was then discarded and added with 250-μL lncRNA XIST probes (300 ng/mL), followed by incubation at 42 °C overnight. After three rinses with PBS-Tween-20 (PBST), the nuclei of the chondrocytes were stained with PBST-diluted DAPI (1:800) in a 24-well plate for 5 min. After three PBST rinses, the chondrocytes were mounted using an anti-fluorescence quenching agent. Finally, images were obtained from five randomly selected fields under a fluorescence microscope (Olympus, Tokyo, Japan).

### Methylation-specific PCR (MS-PCR)

The genomic DNA content of chondrocytes was extracted according to the instructions of the genomic DNA extraction kit (Beijing Tiangen Biochemistry Technology Co., Ltd., Beijing, China). The concentration and purity of DNA were determined with using ultraviolet spectrophotometry and the DNA was stored at − 80 °C. The methylation status of the TIMP-3 promoter was examined by MS-PCR. The DNA was treated with bisulfite, which allowed the unmethylated cytosine residues to convert to uracil, so that the sequences of methylated cytosine would be different from DNA sequencing using different primers to identify the methylation status of DNA [23]. DNA Methylation-Gold™ kits (D5005, Zymo Research, Irvine, CA, USA) were used to detect the methylation level of the TIMP-3 promoter region. TIMP-3 gene primers for methylation and demethylation are shown in Table 2. The purified DNA was used in the subsequent PCR reaction using a reaction column for desulfurization and purification. The reaction products were then analyzed by agarose gel electrophoresis and imaged with a gel electrophoresis imaging analysis system. If the CpG island of the TIMP-3 gene promoter was completely methylated, only the methylated primer could amplify the target band. If there was no methylation at all, only the unmethylated primer could amplify the target band. If it was partially methylated, the target bands could be amplified by both pairs of primers. The experiment was repeated in triplicates to obtain the mean value.

**Table 2:** Primer sequence

Gene	Primer sequence (5′–3′)
TIMP-3-M	F: TCGAGGATTTAGCGGTAAGTATC
	R: GAAAACAAAAAATAACGAAACGAA
TIMP-3-U	F: TTTTGAGGATTTAGTGGTAAGTATTG
	R: CCAAAAACAAAAAATAACAAAACAA

Notes: *F* forward, *R* reverse, *M* methylated, *U* unmethylated, *TIMP-3* tissue inhibitor of metalloproteinase 3

### Chromatin immunoprecipitation (ChIP)

The chondrocytes were fixed in 1% formaldehyde for 10 min at room temperature for cross-linking between the intracellular DNA and protein when the cell confluence reached 70–80%. The cross-linked DNA and protein were randomly broken into fragments of appropriate sizes by ultrasonic treatment. The fragments were centrifuged at 30,237×*g* at 4 °C, after which the supernatant was collected in three tubes, which were supplemented with positive control antibody (RNA polymerase II), NC antibody (normal mouse antibody against IgG, ab10948, dilution ratio of 1:100, Abcam Inc., Cambridge, UK), and the target protein-specific mouse monoclonal antibody to DNA Methyltransferase 1 (DNMT1) (ab13537, dilution ratio of 1:100, Abcam Inc., Cambridge, UK), and rabbit polyclonal antibody to DNMT3A (ab2850, dilution ratio of 1:100, Abcam Inc., Cambridge, UK) and DNMT3B (ab2851, dilution ratio of 1:100, Abcam Inc., Cambridge, UK) for incubation at 4 °C overnight. The endogenous DNA-protein complex was precipitated with Protein Agarose/Sepharose. After transient centrifugation, the supernatant was removed, the nonspecific complex was washed and the cross-link was broken at 65 °C overnight. The DNA fragments were purified and recycled by phenol/chloroform extraction. The enrichment of TIMP-3 promoter fragments binding to DNMT1, DNMT3A, and DNMT3B was tested using specific primers of the TIMP-3 gene promoter region (Table 3) [24].

**Table 3:** Primer sequence

Gene	Primer sequence (5′–3′)
TIMP-3-promoter	F: 5′-CACGGCGGCATTATTCCCTA-3′
	R: 5′-CAATGGCAGAGCCGCATTAC-3′

Note: *F* forward, *R* reverse, *TIMP-3* tissue inhibitor of metalloproteinase 3

### RNA immunoprecipitation (RIP) assay

RIP kits (Merck Millipore, Billerica, MA, USA) were employed to detect the binding of lncRNA XIST to DNMT1, DNMT3A, and DNMT3B protein, respectively. The cells were lysed using a RIPA lysate buffer (P0013B, Beyotime Biotechnology Co., Shanghai, China) for 5 min on an ice bath, with the supernatant collected by means of centrifugation. Afterwards, the cell extracts were incubated with antibodies for co-precipitation. A total of 50-μL magnetic beads were selected for each reaction system of co-precipitation and re-suspension was carried out in 100 μL RIP Wash Buffer. Next, 5 μg antibody was supplemented to the samples for incubation based on grouping. The mixture of magnetic beads and antibody was re-suspended in 900 μL RIP Wash Buffer, and 10 μL cell extracts were added for incubation at 4 °C overnight. Subsequently, the cell samples were placed on a magnet base in order to collect the compound of magnetic beads and antibody. The samples were treated with protease K for RNA extraction, which were stored for subsequent RT-qPCR. The antibodies used for RIP assay, which included the mouse monoclonal antibody to DNMT1 (ab13537, dilution ratio of 1:100, Abcam Inc., Cambridge, UK), and rabbit polyclonal antibody to DNMT3A (ab2850, 1:100, Abcam Inc., Cambridge, UK), and DNMT3B (ab2851, dilution ratio of 1:100, Abcam Inc., Cambridge, UK), were added for incubation at room temperature for 30 min. IgG (ab109489, dilution ratio of 1:100, Abcam Inc., Cambridge, UK) was used as a NC [25].

### RNA pull-down assay

The lncRNA XIST RNA fragments were treated with RNeasy Plus Mini kits (Qiagen, Hilden, Germany). The purified RNA 3′ end was labeled with biotin RNA labeled mixture (Ambion, Austin, Texas, USA). Next, the biotin-labeled RNA (1 μg) was heated with RNA structure buffer (10 mmol/L Tris pH = 7, 0.1 mol/L KCl and 10 mmo/L MgCl_2_) to form the appropriate secondary structure. The chondrocytes (3 μg) were lysed with cell lysis solution (Sigma-Aldrich, St. Louis, MO, USA) for 1 h at 4 °C, and the collected supernatant was transferred to a RNase-free centrifuge tube. Afterwards, 400 ng biotinylated RNA was added to 500 μL RIP buffer and incubation was carried out with cell lysate at room temperature for 1 h. Then, streptavidin beads were added to each binding reaction and incubated at room temperature for 1 h. Finally, the supernatant was rinsed with RIP buffer, supplemented with 5× sample loading buffer and incubated at 95 °C for 5 min. The eluted DNMT1, DNMT3A, and DNMT3B proteins were detected using Western blot analysis.

### Statistical analysis

Statistical analyses were performed using the SPSS 21.0 statistical software (IBM Corp. Armonk, NY, USA). All the data were tested by normal distribution and variance homogeneity test. Measurement data in normal distribution were expressed as mean ± standard deviation, and data between two groups were assessed by *t* test, while data among multiple groups were analyzed using one-way analysis of variance (ANOVA), followed by Tukey’s post hoc test. Data in skewed distribution were analyzed with the use of rank-sum test. A value of *p* < 0.05 was indicative of statistical significance.

## Results

### High expression of lncRNA XIST in cartilage tissues of OA

**Fig. 1:**
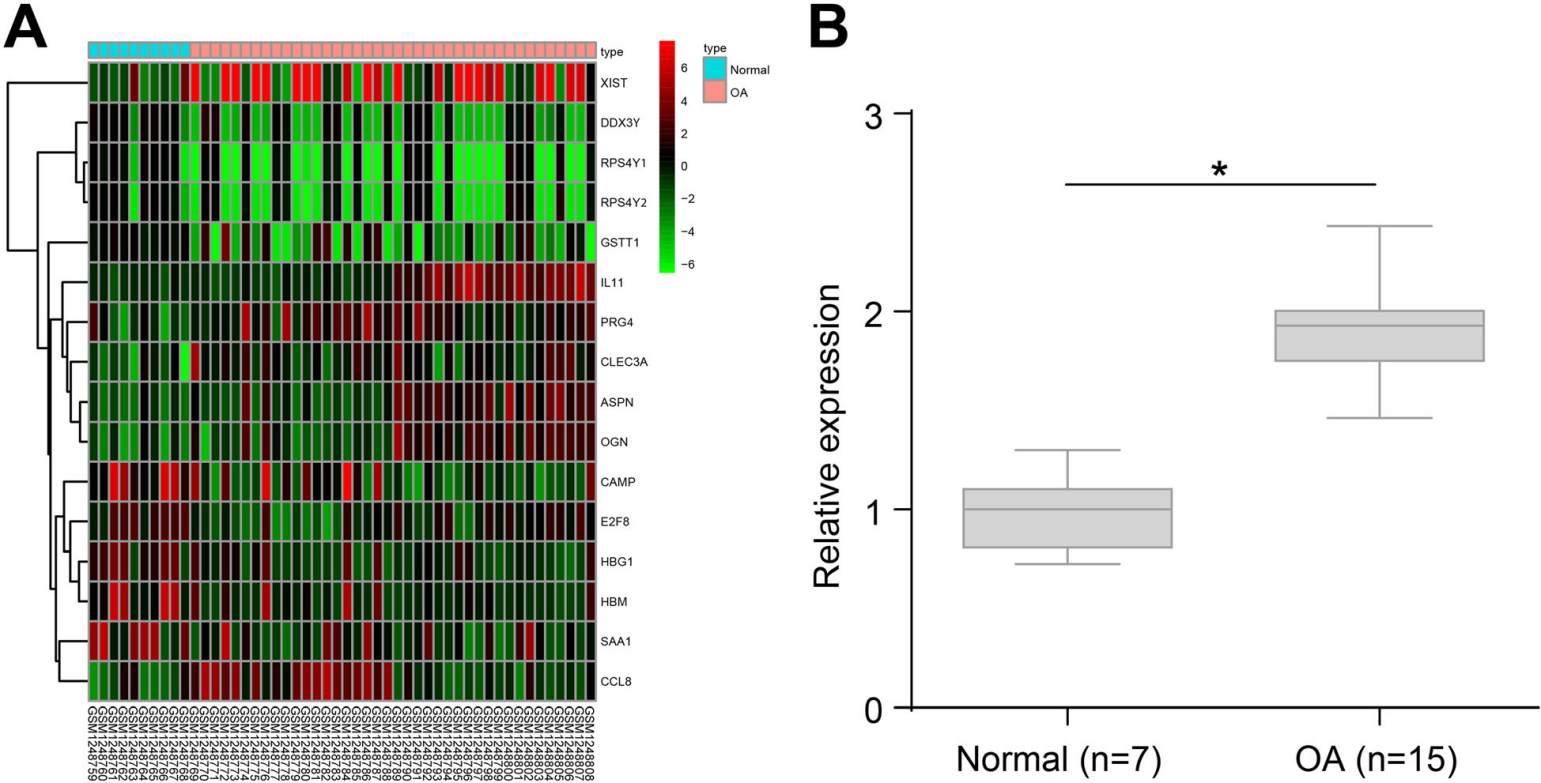
High expression of lncRNA XIST in cartilage tissue of OA. **a** The heatmap of GSE51588 dataset, the horizontal coordinate refers to the sample number, the vertical coordinate refers to the differentially expressed gene, the upper right histogram represents the color gradation, and each rectangle in the diagram corresponds to the expression value of a sample. **b** The expression of lncRNA XIST in normal cartilage tissues and OA cartilage tissues after tibial plateau fracture determined by RT-qPCR; *p < 0.05 vs. the normal cartilage tissues; the measurement data were expressed as the mean ± standard deviation, which were analyzed by the independent sample *t* test; OA cartilage tissue (*n* = 15); normal cartilage tissue (*n* = 7)

Analysis of the gene expression dataset GSE51588 using the R language revealed that lncRNA XIST was highly expressed in OA (Fig. 1a). In order to further explore the relationship between lncRNA XIST and OA, we examined the expression of lncRNA XIST in normal cartilage and OA cartilage tissues following a tibial plateau fracture with RT-qPCR. The results demonstrated that OA cartilage tissues exhibited higher expressions of lncRNA XIST compared to normal cartilage tissues (Fig. 1b).

### Successful isolation and identification of chondrocytes

**Fig. 2:**
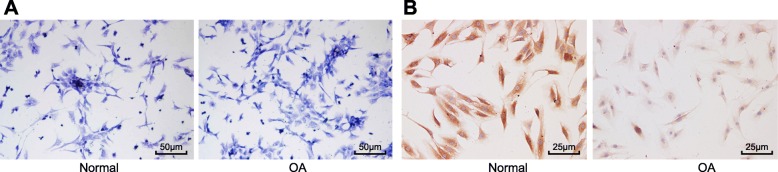
Successful isolation and culture of chondrocytes. **a** Normal and OA cartilage tissues stained with toluidine blue (× 200). **b** Immunocytochemical staining of normal and OA chondrocytes (× 400)

The chondrocytes were successfully isolated from normal cartilage and OA cartilage tissues and stained with toluidine blue staining for identification. As shown in Fig. 2a, toluidine blue staining illustrated that the chondrocytes were fusiform-shaped in normal cartilage tissues with dark blue nuclei, whereas there was a change in the chondrocytes from OA cartilage tissues, which appeared to have long-fusiform or irregular shapes. In addition, the color of cytoplasm and nucleus was relatively lighter compared to that in the normal cartilage tissues. Furthermore, we applied immunocytochemical staining to detect type II collagen (Fig. 2b), which demonstrated that type II collagen was positively stained as yellowish-brown coloration in normal chondrocytes, while the lighter color of OA chondrocytes indicated their weakly positive nature. Overall, the abovementioned findings demonstrated the successful isolation of chondrocytes from normal and OA cartilage tissues.

### Silencing lncRNA XIST inhibits collagen degradation in chondrocytes of OA

**Fig. 3:**
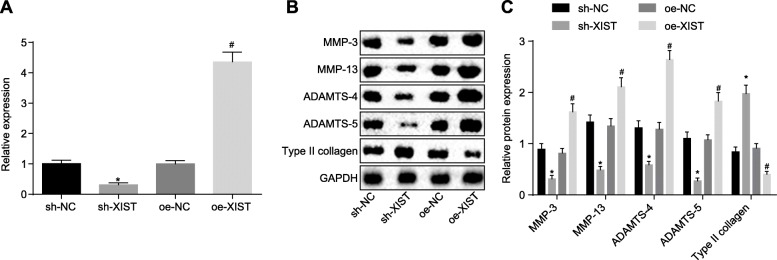
Silencing lncRNA XIST suppresses the degradation of collagen in chondrocyte of OA. **a** Expression of lncRNA XIST in cells transfected with pGPU6/Neo-XIST-XIST or pCMV6-AC-GFP-XIST determined by RT-qPCR. **b**, **c** Expression of MMP-3, MMP-13, ADAMTS-4, ADAMTS-5, and type II collagen protein determined by Western blot analysis; * *p* < 0.05 vs. the sh-NC group (cells transfected with pGPU6/Neo); # *p* < 0.05 vs. the oe-NC group (cells transfected with pCMV6-AC-GFP); the measurement data were expressed as the mean ± standard deviation, which were analyzed by one-way analysis of variance; the experiment was repeated three times to obtain the mean value

After successfully isolating chondrocytes from normal and OA cartilage tissues, we determined the expression of lncRNA XIST and related proteins in OA chondrocytes treated with either silencing or over-expression of lncRNA XIST in order to elucidate the effect of lncRNA XIST on OA. The efficiency of plasmid transfection in cells transfected with pGPU6/Neo-XIST or pCMV6-AC-GFP-XIST was measured using RT-qPCR (Fig. 3a). The results revealed that the expressions of lncRNA XIST were significantly decreased in cells transfected with pGPU6/Neo-XIST, while being increased in cells transfected with pCMV6-AC-GFP-XIST, suggesting that transfection efficiency met the requirements for subsequent experimentation. In addition, Western blot analysis was employed to determine the expression of the related proteins (Fig. 3b, c). The findings demonstrated a significant decrease in the expressions of MMP-3, MMP-13, ADAMTS-4, and ADAMTS-5 protein, while that of type II collagen protein was elevated in cells undergoing silencing of lncRNA XIST, whereas opposite trends were observed in cells with over-expressed lncRNA XIST. These results indicated that lncRNA XIST affected the degradation of collagen in chondrocytes of OA, and silencing lncRNA XIST resulted in the inhibition of collagen degradation in chondrocytes of OA.

### TIMP-3 inhibits collagen degradation in chondrocytes of OA

**Fig. 4:**
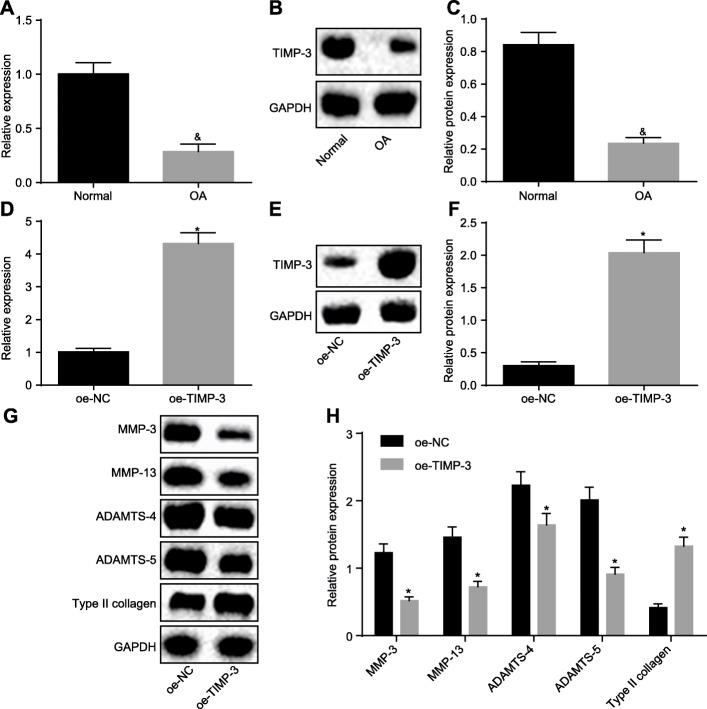
Over-expression of TIMP-3 inhibits the degradation of collagen in chondrocytes of OA. **a** RT-qPCR used to detect the mRNA expression of TIMP-3 in OA cartilage tissues and normal cartilage tissues determined by RT-qPCR. **b**, **c** The protein expression of TIMP-3 in OA cartilage tissues and normal cartilage tissues determined by Western blot analysis. **d** The mRNA expression of TIMP-3 in cells transfected with pCMV6-AC-GFP-TIMP-3 plasmids determined by RT-qPCR. **e**, **f** The mRNA expression of TIMP-3 in cells transfected with pCMV6-AC-GFP-TIMP-3 plasmids determined by Western blot analysis. **g**, **h** The expression of related proteins (MMP-3, MMP-13, ADAMTS-4, ADAMTS-5, and type II collagen) determined by Western blot analysis; & *p* < 0.05 vs. the normal group (normal cartilage tissue); * *p* < 0.05 vs. the oe-NC group (cells transfected with pCMV6-AC-GFP); the measurement data were expressed as the mean ± standard deviation, which were analyzed by the independent sample *t* test; the experiment was repeated three times to obtain the mean value; OA cartilage tissue (*n* = 15); normal cartilage tissue (*n* = 7)

TIMPs are natural inhibitors of MMPs. TIMP-3 belongs to the TIMPs family, and a previous study revealed that TIMP-3 deficiency can lead to mild cartilage degeneration in patients with OA [26]. In view of this, we employed RT-qPCR and Western blot analysis to determine the expression of TIMP-3 in normal cartilage tissues and OA cartilage tissues (Fig. 4a–c). The results demonstrated that the expression of TIMP-3 in OA cartilage tissues was significantly lower compared to the normal cartilage tissues (*p* < 0.05). In order to further investigate the effect of TIMP-3 on OA, we treated chondrocytes of OA with TIMP-3 over-expression plasmids, and detected plasmid transfection efficiency in cells transfected with pCMV6-AC-GFP-TIMP-3 or pCMV6-AC-GFP with RT-qPCR and Western blot analysis (Fig. 4d–f). The results demonstrated that the expression of TIMP-3 was higher in cells transfected with pCMV6-AC-GFP-TIMP-3, indicating that the efficiency of transfection met the requirements for subsequent experimentation. Subsequently, Western blot analysis was applied to determine the expression of the related proteins (Fig. 4g, h). The results showed a remarkable decrease in the expressions of MMP-3, MMP-13, ADAMTS-4, and ADAMTS-5 protein, while that of type II collagen protein increased significantly in cells with over-expressed TIMP-3. These findings led to the conclusion that TIMP-3 could affect the degradation of collagen in chondrocytes of OA, and over-expression of TIMP-3 could lead to the inhibition of collagen degradation in chondrocytes of OA.

### LncRNA XIST promotes TIMP-3 promoter methylation by recruiting DNMT1, DNMT3A, and DNMT3B to TIMP-3 promoter

**Fig. 5:**
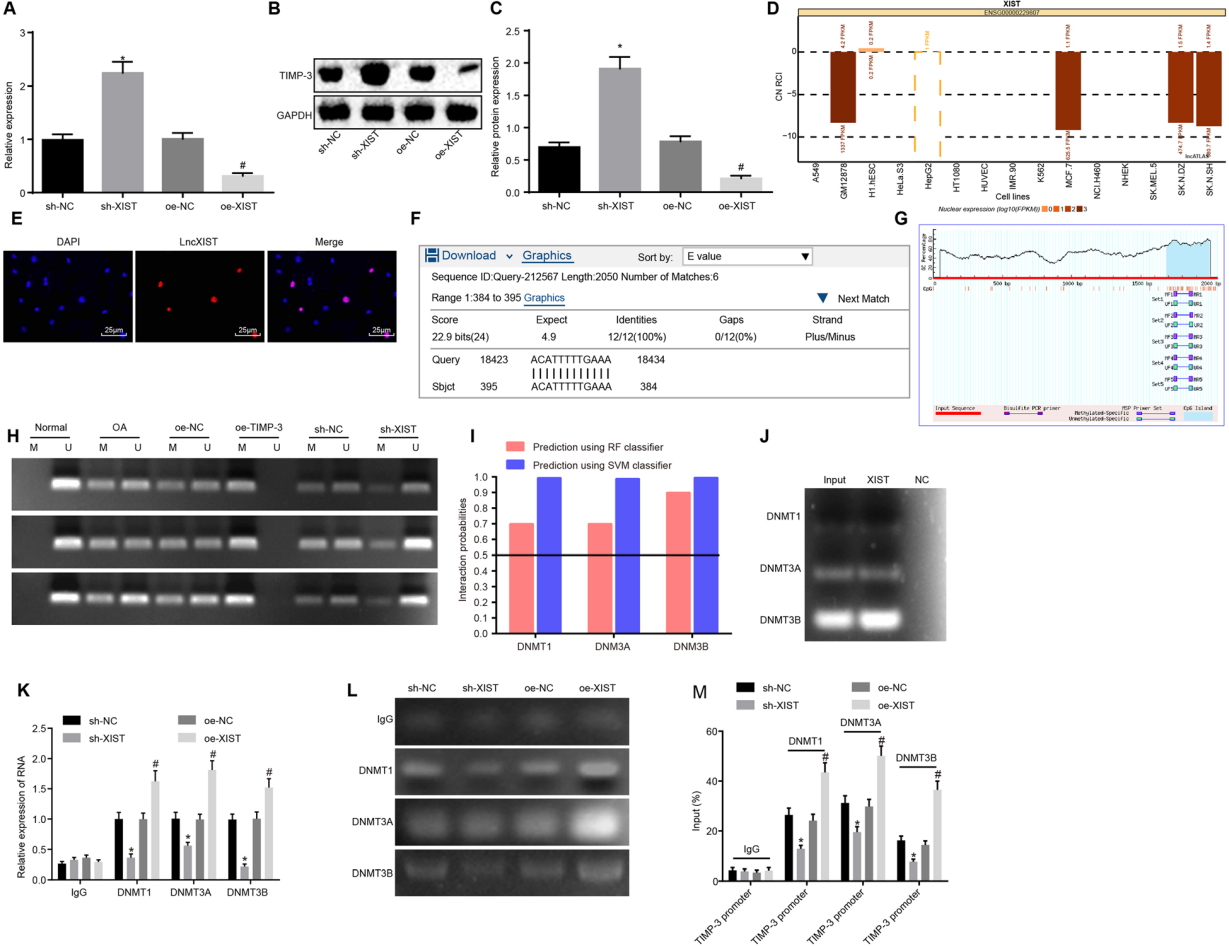
LncRNA XIST inhibits the expression of TIMP-3 by recruiting DNA methyltransferases into the TIMP-3 promoter region. **a** The mRNA expression of TIMP-3 after silencing and over-expression of lncRNA XIST determined by RT-qPCR. **b**, **c** The protein expression of TIMP-3 after silencing and over-expression of lncRNA XIST determined by Western blot analysis. **d** Prediction of subcellular localization of lncRNA XIST available on lncATLAS website. **e** Detection of subcellular localization of lncRNA XIST in OA chondrocytes by FISH assay (× 400). **f** Blast alignment between lncRNA XIST sequence and TIMP-3 promoter sequence. **g** Prediction of CpG island enrichment in TIMP-3 promoter region by MethPrimer website. **h** Determination of methylation status of TIMP-3 promoter in each group using different primers by MS-PCR assay. **i** Prediction of combining ability between lncRNA XIST sequence and DNA methyltransferase DNMT1, DNMT3A, and DNMT3B protein sequences, and the combining ability is reflected by when random forest (RF) > 0.5 and support vector machine (SVM) > 0.5. **j** The binding of lncRNA XIST to DNMT1, DNMT3A, and DNMT3B measured by RNA pull-down assay. **k** RIP assay used to detect the enrichment of lncRNA XIST by DNMT1, DNMT3A, and DNMT3B, and the amount of enriched lncRNA XIST enriched detected by RT-qPCR. **l**, **m** The enrichment of DNMT1, DNMT3A, and DNMT3B in TIMP-3 promoter detected by ChIP, and TIMP-3 promoter fragment is measured by RT-qPCR; * *p* < 0.05 vs. the sh-NC group (cells transfected with pGPU6/Neo); #, *p* < 0.05 vs. the oe-NC group (cells transfected with pCMV6-AC-GFP); the measurement data were expressed as the mean ± standard deviation, which were analyzed by the independent sample *t* test or one-way analysis of variance; the experiment was repeated three times to obtain the mean value

In order to investigate whether lncRNA XIST and TIMP-3 exerted a certain regulatory mechanism in OA, we examined the expression of TIMP-3 in each group following silencing and over-expression of lncRNA XIST in chondrocytes (Fig. 5a–c). The findings demonstrated that the expression of TIMP-3 was significantly decreased in cells transfected with pCMV6-AC-GFP-XIST, while being evidently increased in cells transfected with pGPU6/Neo-XIST, indicating that there might be a correlation between lncRNA XIST and TIMP-3 in the progression of OA. To investigate the regulatory mechanism between lncRNA XIST and TIMP-3, we applied the lncATLAS website (http://lncatlas.crg.eu/) and found that lncRNA XIST was primarily localized in the nucleus (Fig. 5d), which was further confirmed by means of FISH assay (Fig. 5e), indicating that it might possess the function of transcriptional regulation. Furthermore, Blast online alignment revealed that lncRNA XIST might bind to the TIMP-3 promoter in the form of RNA-DNA (Fig. 5f). In addition, the presence of CpG islands in the TIMP-3 promoter region (Fig. 5g) was predicted by the MethPrimer (http://www.urogene.org/cgi-bin/methprimer/methprimer.cgi) website, suggesting that there was a CpG island in the TIMP-3 promoter region. In order to verify this prediction, chondrocytes were isolated form three normal cartilage tissue samples and three OA cartilage tissue samples. A portion of cells from OA cartilage tissue samples were treated with plasmids of oe-NC, oe-XIST, sh-NC, and sh-XIST. After being treated, MS-PCR was conducted on chondrocytes to assess the methylation status of TIMP-3 promoter in each group (Fig. 5h). No methylation was observed in the CpG islands of the TIMP-3 promoter region in normal chondrocytes, while partial methylation was detected in OA chondrocytes. Moreover, exhaustive methylation was apparent in the CpG islands of the TIMP-3 promoter region after lncRNA XIST was over-expressed in OA chondrocytes. In addition, the methylation degree was found to be higher in lncRNA XIST-over-expressed cells, while lower in cells with silenced lncRNA XIST when compared with the corresponding controls, respectively. All in all, these findings demonstrated that lncRNA XIST inhibited TIMP-3 transcription by increasing the methylation level of the TIMP-3 promoter.

Additionally, RPIseq database (http://pridb.gdcb.iastate.edu/RPISeq/) analysis revealed that lncRNA XIST might bind to the DNMT1, DNMT3A, and DNMT3B genes (Fig. 5i). Subsequently, RNA pull-down assay was utilized to explore the binding between lncRNA XIST and DNA methyltransferases (Fig. 5j). The results revealed that lncRNA XIST pulled-down all DNMT1, DNMT3A, and DNMT3B. Then, RIP assay was performed to analyze the enrichment of lncRNA XIST by DNMT1, DNMT3A, and DNMT3B (Fig. 5k). Results demonstrated that following the over-expression of lncRNA XIST, the enrichment of lncRNA XIST on DNMT1, DNMT3A, and DNMT3B all increased significantly, while decreasing significantly after lncRNA XIST silencing. In addition, ChIP was performed to further verify the enrichment of DNMT1, DNMT3A, and DNMT3B in the TIMP-3 promoter (Fig. 5l–m). It was found that following the over-expression of lncRNA XIST, the enrichment of DNMT1, DNMT3A, and DNMT3B all significantly increased in the TIMP-3 promoter, while being evidently decreased following lncRNA XIST silencing. These results suggested that lncRNA XIST inhibited the expression of TIMP-3 by recruiting DNA methyltransferases into the TIMP-3 promoter region, while inhibiting the transcription of TIMP-3.

### Over-expression of TIMP-3 reverses the effect of lncRNA XIST on collagen degradation in chondrocytes

**Fig. 6:**
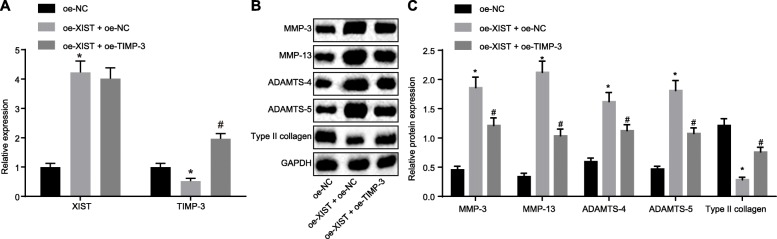
Over-expression of TIMP-3 reverses the effect of lncRNA XIST on collagen degradation in chondrocytes. **a** Relative expression of lncRNA XIST and mRNA expression of TIMP-3 determined by RT-qPCR. **b**, **c** The expression of MMP-3, MMP-13, ADAMTS-4, ADAMTS-5, and type II collagen determined by Western blot analysis. * *p* < 0.05 vs. the oe-NC group (cells transfected with pCMV6-AC-GFP); # *p* < 0.05 vs. the oe-XIST + oe-NC group (cells transfected with pCMV6-AC-GFP-XIST + pCMV6-AC-GFP); the measurement data were expressed as the mean ± standard deviation, which were analyzed by one-way analysis of variance; the experiment was repeated three times

To further investigate the effect of lncRNA XIST and TIMP-3 on OA, OA chondrocytes were treated with pCMV6-AC-GFP-XIST + pCMV6-AC-GFP, pCMV6-AC-GFP-XIST + pCMV6-AC-GFP-TIMP-3, and pCMV6-AC-GFP. The expression of lncRNA XIST and mRNA expression of TIMP-3 were determined by RT-qPCR (Fig. 6a). The results revealed a significant increase in lncRNA XIST expression when cells were treated with pCMV6-AC-GFP-XIST + pCMV6-AC-GFP, while TIMP-3 expression decreased compared to the treatment of pCMV6-AC-GFP. The transfection of pCMV6-AC-GFP-XIST + pCMV6-AC-GFP-TIMP-3 led to higher TIMP-3 expression, while the lncRNA XIST expression did not differ significantly in comparison with the treatment of CMV6-AC-GFP-XIST + pCMV6-AC-GFP. In addition, Western blot analysis was performed to determine the expression of related proteins (MMP-3, MMP-13, ADAMTS-4, ADAMTS-5, and type II collagen) (Fig. 6b, c). The results demonstrated that the expressions of MMP-3, MMP-13, ADAMTS-4, and ADAMTS-5 protein were significantly increased in cells treated with pCMV6-AC-GFP-XIST + pCMV6-AC-GFP, while the expression of type II collagen protein was significantly decreased compared to cells with pCMV6-AC-GFP. The transfection of pCMV6-AC-GFP-XIST + pCMV6-AC-GFP-TIMP-3 induced opposite results when compared to the treatment of CMV6-AC-GFP-XIST + pCMV6-AC-GFP. These findings suggested that over-expression of TIMP-3 could reverse the effect of lncRNA XIST on collagen degradation in chondrocytes of OA.

## Discussion

Researchers have extensively explored the changes in extracellular matrix in OA, such as MMPs, collagen, and proteoglycan; however, the underlying mechanism of OA pathogenesis remains unclear [27]. Fortunately, all efforts have not been in vain as recent findings have identified lncRNAs as one of the chief contributors to OA pathology attributable to their regulatory role in chondrocyte proliferation, apoptosis, and autophagy [28–30]. The current study aimed to explore the effect of lncRNA XIST on collagen degradation in chondrocytes of OA after tibial plateau fracture by modulating TIMP-3 promoter methylation. Collectively, our findings illustrated that lncRNA XIST increases the methylation of the TIMP-3 promoter by recruiting DNA methyltransferase to TIMP-3 promoter and inhibits the transcription of TIMP-3, thereby promoting collagen degradation in chondrocytes of OA after tibial plateau fracture.

Firstly, in silico analyses revealed that OA exhibits high expressions of lncRNA XIST, which was further verified by comparing OA cartilage tissues and normal cartilage tissues. A prior study also demonstrated that lncRNA XIST is significantly upregulated in OA cartilage tissues, and even regarded lncRNA XIST as a potential therapeutic biomarker for OA treatment [12]. Meanwhile, our findings confirmed that lncRNA XIST was an upstream regulatory gene of TIMP-3, and TIMP-3 was downregulated in OA cartilage tissues in contrast to normal cartilage tissue. Another study, which primarily focused on the differences between aggrecan and type II collagen turnover, suggested that TIMP-3-null mice exhibited a higher degree of cartilage degradation compared to wild-type ones [26]. The aforementioned results highlighted the vital role of lncRNA XIST and TIMP-3 in the progression of OA.

Our study also uncovered that lncRNA XIST induced collagen degradation in OA chondrocytes after the occurrence of tibial plateau fracture, which was achieved by promoting TIMP-3 promoter methylation through enrichment of DNA methyltransferase to TIMP-3 promoter and decreasing the transcription of TIMP-3. It has been reported that aggrecan degradation is induced by the action of MMPs and ADAMT families [31]. TIMPs have also been suggested to possess the ability to suppress the members of two other MMP families, namely the ADAMs and the ADAMTs [32], wherein TIMP-3 plays an inhibitory role to ADAMTS-1, ADAMTS-4, ADAMTS-5, ADAMTS-8, ADAMTS-9, and ADAMTS-15 [33]. In line with our observations, a previous study also documented slightly elevated rates of TIMP-3 endocytosis in the presence of proMMP-13, in addition to slightly reduced rates of proMMP-13 endocytosis in the presence of TIMP-3 [34]. Furthermore, a function study reported that the S100A8 stimulation in chondrocytes induces upregulation of mRNA levels of MMP-2, MMP-3, MMP-9, and MMP-13 and ADAMTS-4 and ADAMTS-5 in experimental murine arthritis, which is similar to our findings [35]. ADAMTS-4 and ADAMTS-5 are two prominent degradation factors in joint structure [36]. Furthermore, studies have also shown that the loss of aggrecan is a key event in early OA with the actions of aggrecanase enzymes, and ADAMTS-4 and ADAMTS-5 which has been identified as the main cartilage aggrecanases in humans [37]. However, the exact role of lncRNA XIST in OA still awaits further investigations. Moreover, future studies are warranted on the regulatory relationship between TIMP-3 and aggrecans. A recent study mentioned that lncRNA XIST is the only gene exclusively expressed from the inactive X chromosome, and some genes are normally subject to inactivation of X chromosome, including TIMP-1 from the short arm of the X chromosome [38]. DNMT family comprises of conserved DNA-modifying enzymes responsible for epigenetic gene regulation, including transcription activation, transcriptional silencing, and post-transcriptional regulation [39]. In addition, DNMT1 specifically encodes the maintenance methyltransferase, while DNMT3A or DNMT3B encodes the de novo methyltransferase [40]. Our findings illustrated that lncRNA XIST inhibits the transcription of TIMP-3 to downregulate the expression of TIMP-3 through the rapid recruitment of maintenance DNA methyltransferase DNMT1 and inducing the amount of de novo methyltransferases DNMT3A and DNMT3B, which further gather and bind in the TIMP-3 promoter region to increase the methylation ratio of its CpG island. Downregulation of TIMP-3 could promote collagen degradation in chondrocytes of OA after the occurrence of tibial plateau fracture. In conclusion, lncRNA XIST and TIMP-3 form an epigenetic axis to regulate collagen activity.

## Conclusion

**Fig. 7:**
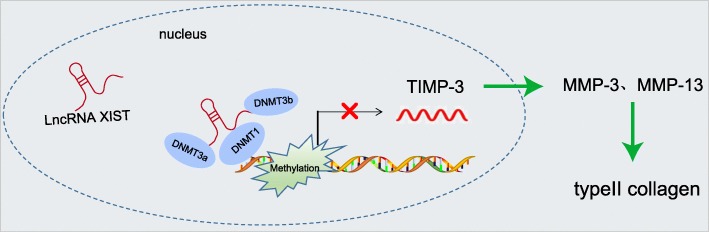
The mechanism investigation indicates that lncRNA XIST is a novel significant regulator of the development of OA by raising the methylation of TIMP-3 promoter, thus promoting collagen degradation in chondrocytes of OA after tibial plateau fracture

Overall, the current study uncovered that lncRNA XIST regulates OA development by raising the methylation of TIMP-3 promoter, thus promoting collagen degradation in chondrocytes of OA following tibial plateau fracture (Fig. 7). Our findings shed a new light on the recognition of the pathogenic role of lncRNA XIST and TIMP-3 in the progression of OA, which suggests that both the molecules may be promising targets for the treatment of patients with OA. However, further studies are required to confirm our findings in in vivo experiments.

## Data Availability

The datasets generated/analyzed during the current study are available.

## Abbreviations

ANOVA: Analysis of variance
BCA: Bicinchoninic acid
DEGs: Differentially expressed genes
ECL: Enhanced chemiluminescence
ECM: Extracellular matrix
GEO: Gene Expression Omnibus
lncRNAs: Long noncoding RNAs
OA: Osteoarthritis
PMSF: Phenylmethylsulfonyl fluoride
PVDF: Polyvinylidene fluoride
SDS-PAGE: Sodium dodecyl sulfate polyacrylamide gel electrophoresis
TIMP-3: Tissue inhibitor of metalloproteinase 3
XIST: X-inactive specific transcript
